# Postoperative circulating tumor DNA in stage II colon cancer: biological rationale, clinical evidence, and unresolved challenges

**DOI:** 10.3389/fonc.2026.1908802

**Published:** 2026-09-11

**Authors:** Erhua Yang, Xuefeng Wang, Jiajing Zhang, Dejiang Zhao, Zhimeng Yang, Xue Li, Guirong He, Tianhua Guo, Ruotian Wang, Baohong Yuan

**Affiliations:** 1 Department of General Surgery, Yan’An Hospital Affiliated to Kunming Medical University, Kunming, China; 2 Nujiang People’s Hospital, Nujiang, China; 3 The Key Laboratory of Tumour Immunological Prevention and Treatment of Yunnan Province, Kunming Medical University, Kunming, China

**Keywords:** adjuvant chemotherapy, circulating tumor DNA, liquid biopsy, minimal residual disease, risk stratification, stage II colon cancer

## Abstract

**Background:**

Stage II colon cancer is clinically heterogeneous. Current clinicopathologic risk factors cannot accurately identify patients with residual disease after curative resection. Circulating tumor DNA (ctDNA) has emerged as a promising biomarker for the detection of minimal residual disease (MRD), defined as microscopic residual tumor burden that remains after curative-intent treatment and is not detectable by conventional imaging, and for postoperative risk stratification.

**Methods:**

This narrative review summarizes the biological basis, analytical approaches, and clinical evidence regarding ctDNA in stage II colon cancer. We particularly emphasize prospective studies and randomized controlled trials.

**Results:**

Postoperative ctDNA positivity is strongly associated with increased recurrence risk and provides superior prognostic stratification compared with conventional clinicopathologic factors. Prospective studies have demonstrated that ctDNA-positive patients experience substantially higher recurrence rates, with the GALAXY study reporting a hazard ratio of 11.99 (95% CI: 8.83–16.27) for disease-free survival among patients with postoperative molecular residual disease. The DYNAMIC trial demonstrated that ctDNA-guided management reduces adjuvant chemotherapy use without compromising recurrence-free survival. However, current evidence suggests an important asymmetry in clinical utility. ctDNA negativity may support treatment de-escalation. In contrast, ctDNA positivity has not yet reliably identified patients who benefit from treatment escalation.

**Conclusions:**

ctDNA constitutes a robust prognostic biomarker in stage II colon cancer and supports risk-adapted postoperative management. However, its predictive value for guiding treatment escalation remains unproven. Integration with clinicopathologic and molecular features is essential before routine clinical implementation.

## 1 Introduction

Colorectal cancer remains a major cause of cancer-related morbidity and mortality worldwide (1). Among patients with non-metastatic disease, approximately one-third present with stage II colon cancer, a group generally associated with favorable outcomes following curative-intent resection. Nevertheless, postoperative recurrence still occurs in approximately 10–20% of patients with stage II colon cancer after curative-intent surgery, including a subset of patients classified as low risk according to conventional clinicopathologic criteria. This indicates the presence of occult residual disease that is not adequately captured by current staging systems and highlights the unmet need for more precise postoperative risk stratification (2). Although five-year survival for stage II colon cancer is substantially better than for stage III disease, the central clinical challenge lies in identifying the minority of patients whose recurrence risk is high enough to justify postoperative systemic therapy (3).

The role of adjuvant chemotherapy in stage II colon cancer remains controversial (4). Randomized studies have shown only modest absolute benefit at the population level (5), and current clinical decision-making therefore relies largely on clinicopathologic high-risk features, including T4 stage, lymphovascular invasion, perineural invasion, poor differentiation, bowel obstruction or perforation, and examination of fewer than 12 lymph nodes (6, 7). However, these variables are primarily prognostic rather than predictive in nature and do not directly quantify residual tumor burden (8). In addition, molecular features such as mismatch repair deficiency (dMMR) or microsatellite instability-high (MSI-H) status substantially influence prognosis and treatment relevance in stage II disease, underscoring the limitations of a purely morphology-based risk model (9, 10). Circulating tumor DNA (ctDNA) has emerged as a promising biomarker for the molecular detection of MRD after curative-intent surgery (11). ctDNA refers to the tumor-derived fraction of circulating cell-free DNA (cfDNA) and can be detected in plasma using highly sensitive sequencing-based assays (12). Because ctDNA has a short half-life, postoperative ctDNA detection can provide a near real-time indication of residual disease (13). In colorectal cancer, multiple studies have shown that postoperative ctDNA positivity is strongly associated with recurrence risk (14, 15), and recent prospective trials have begun to test whether ctDNA-guided treatment strategies can reduce overtreatment or improve risk-adapted care (16, 17).

Importantly, current evidence reveals an important asymmetry in clinical application. Postoperative ctDNA negativity can support treatment de-escalation with increasing confidence. However, ctDNA positivity does not yet reliably identify patients who benefit from treatment escalation. This distinction has critical implications for clinical decision-making and is frequently underappreciated in the literature.

In this review, we contend that ctDNA should currently be interpreted as a de-escalation-enabling biomarker rather than a treatment-escalation determinant, and we propose an integrated clinical framework to contextualize its application alongside established risk factors. Key unresolved issues include biological heterogeneity in ctDNA shedding, false-negative results in low-volume disease, variability across analytical platforms, uncertainty regarding the optimal timing of sampling, and limited evidence supporting treatment escalation in ctDNA-positive patients (18, 19). Different ctDNA detection platforms, including tumor-informed and tumor-uninformed (also referred to as tumor-agnostic or tumor-agnostic) approaches, differ substantially in sensitivity, specificity, and susceptibility to clonal hematopoiesis (20, 21). Furthermore, there is currently no consensus on the optimal timing for post-surgical sampling (4–10 weeks post-surgery), with substantial variation in time windows across studies (22). A biomarker with strong prognostic value does not necessarily predict treatment benefit. Prognostic and predictive functions should therefore be interpreted separately (23).

**Figure 1:**
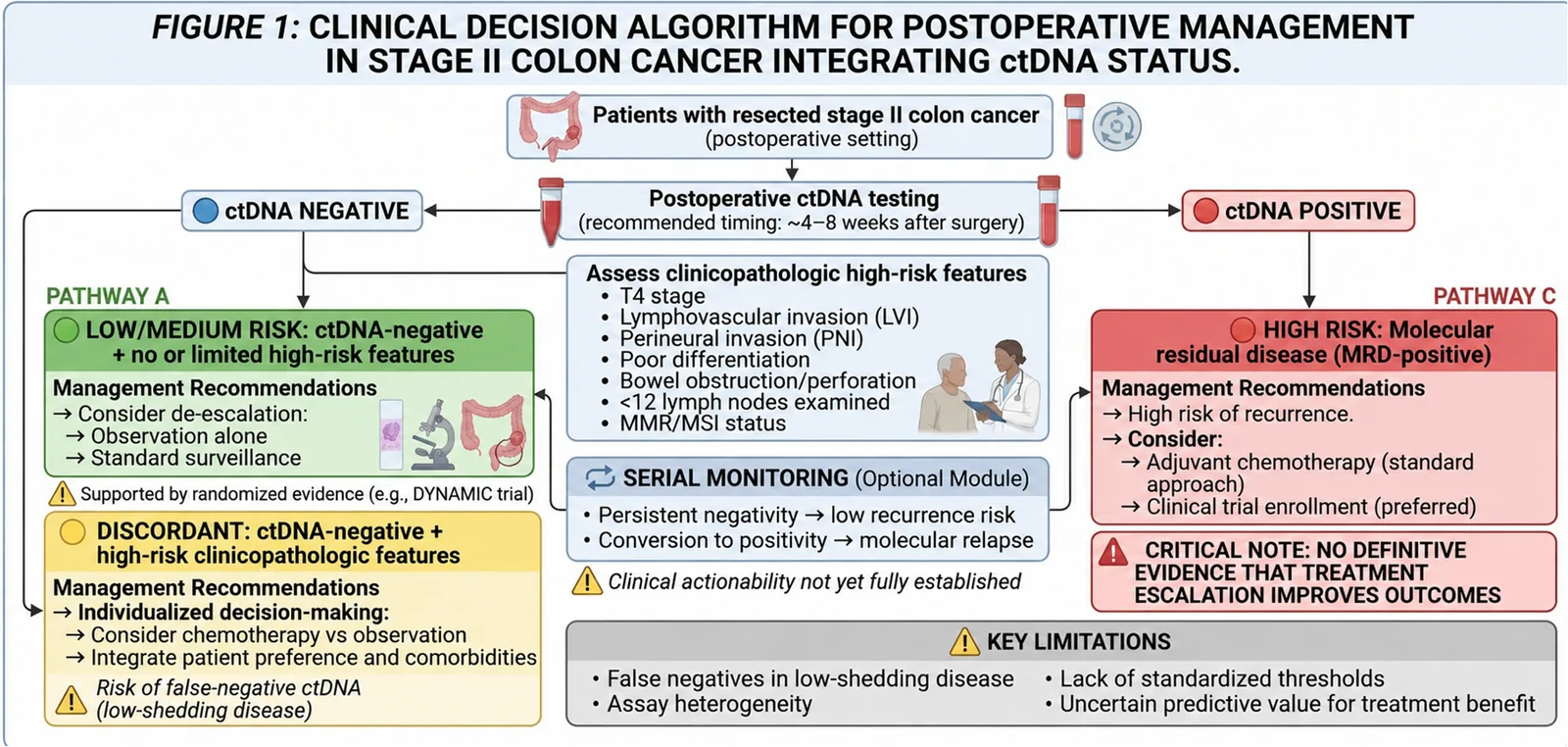
Conceptual decision-support framework for postoperative management in stage II colon cancer integrating ctDNA status.

In this review, we critically examine the biological basis of ctDNA, summarize the current evidence for postoperative ctDNA testing in stage II colon cancer, compare ctDNA with established clinicopathologic and molecular risk factors, and discuss the key challenges that must be addressed before routine clinical implementation can be justified (24, 25). We propose a conceptual decision-support framework (**Figure 1**) for postoperative management. This framework integrates ctDNA status with clinicopathologic risk factors and molecular features to assist adjuvant treatment decisions.

The algorithm is organized into distinct clinical pathways based on concordant or discordant risk profiles and will serve as a reference throughout the review to identify where current evidence is sufficient—and where it remains critically deficient.

## 2 Review strategy

A structured literature search was conducted in PubMed, Embase, and ClinicalTrials.gov from database inception to March 2026 using combinations of the terms “ctDNA,” “circulating tumor DNA,” “colon cancer,” “stage II,” and “minimal residual disease.” Eligible studies included prospective cohorts, randomized controlled trials, and high-quality observational studies focusing on postoperative ctDNA detection, prognostic significance, and ctDNA-guided treatment strategies in stage II colon cancer. Relevant references from selected articles and recent reviews were also screened. Studies lacking clinical relevance, small retrospective studies, and non-peer-reviewed reports were excluded unless necessary for conceptual discussion. As this was a narrative review, no formal meta-analysis was performed; however, the literature identification and selection process were documented to enhance transparency and reproducibility.

## 3 Biological basis of ctDNA and the conceptual framework of MRD

Circulating tumor DNA represents the tumor-derived fraction of cfDNA detectable in plasma (12). Under physiological conditions, cfDNA is released predominantly from hematopoietic cell turnover (26). In patients with cancer, a small proportion of circulating DNA fragments carries tumor-specific genomic or epigenomic alterations, including somatic mutations, copy number changes, and aberrant methylation patterns, thereby defining ctDNA (27). These fragments often exhibit a characteristic size distribution consistent with nucleosomal fragmentation and may be shorter than cfDNA derived from non-malignant tissues, a feature increasingly exploited in fragmentomic approaches to enhance assay sensitivity (28, 29).

**Figure 2:**
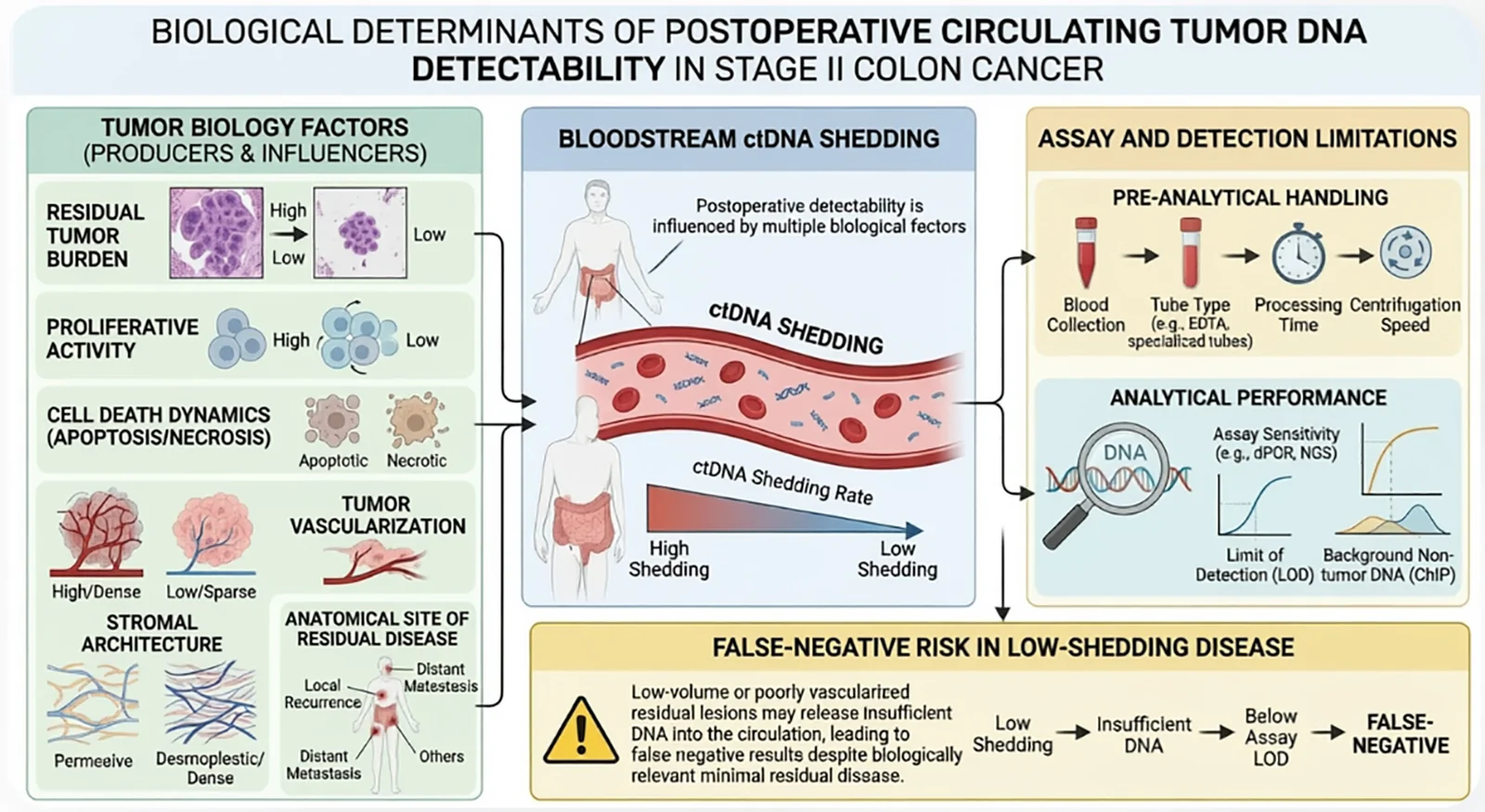
Biological determinants of postoperative ctDNA detectability in stage II colon cancer.

The mechanisms underlying ctDNA release remain incompletely understood. Multiple biological processes are likely involved (30). Apoptosis is considered a major source of ctDNA. Necrosis may also contribute larger and more heterogeneous DNA fragments, particularly in rapidly proliferating or hypoxic tumors (31). Additional mechanisms, such as active DNA release through extracellular vesicles, have also been proposed. However, their quantitative contribution in human tumors remains uncertain (32). Importantly, ctDNA shedding is not uniform across patients or tumor types. ctDNA shedding is influenced by multiple biological factors. These include tumor burden, proliferative activity, vascularization, stromal architecture, anatomical site, and metastatic niche characteristics (33). **Figure 2** synthesizes these key biological determinants of ctDNA detectability, illustrating how tumor-intrinsic factors (e.g., shedding efficiency, proliferative index), tumor-extrinsic factors (e.g., vascularity, anatomical location), and technical assay parameters collectively determine whether residual disease yields a positive ctDNA signal.

This biological heterogeneity is particularly relevant in stage II colon cancer, where residual disease burden is often very low and some tumors may shed insufficient DNA to be consistently detectable (19).

The short half-life of ctDNA supports its use as a dynamic biomarker of tumor burden (13). After curative-intent resection, ctDNA levels are expected to decline rapidly, and persistent postoperative detection is commonly interpreted as molecular evidence of residual disease (15). However, ctDNA should be regarded as an indirect marker of residual disease rather than a direct measure of viable tumor cells (34). Undetectable ctDNA does not completely exclude persistent micrometastatic disease. False-negative results may occur in low-shedding tumors, dormant disease, or poorly vascularized sites such as peritoneal surfaces (35). Accordingly, ctDNA is best understood as a probabilistic molecular indicator associated with MRD rather than as a binary surrogate of its presence or absence (36).

Two major analytical approaches are currently used for ctDNA detection: tumor-informed and tumor-agnostic strategies. Several commercially available platforms have been developed based on these principles (**Table 1**). However, reported performance characteristics should be interpreted cautiously because differences in assay design, patient populations, sampling schedules, and validation endpoints limit direct comparison across platforms.

**Table 1:** Representative commercially available ctDNA platforms and their characteristics.

Platform	Company	Assay type	Tumor tissue required	Key features	Reported performance*	Regulatory status
Signatera™	Natera	Tumor-informed	Yes	Personalized assay based on tumor-specific variants; widely used in colorectal cancer MRD studies	High specificity; sensitivity varies by disease burden and sampling time	Used clinically as a laboratory-developed test (LDT); regulatory status varies by region
Guardant Reveal®	Guardant Health	Tumor-agnostic	No	Plasma-only assay integrating genomic and epigenomic signals	Designed for tissue-free MRD detection; performance varies across studies	Commercially available; regulatory status varies by indication
Tempus xM / xM MRD-related assays	Tempus	Tumor-informed / hybrid approaches	Usually required	NGS-based personalized monitoring approaches	Platform-dependent analytical performance	Commercially available; regulatory status depends on application
SafeSeqS-based assays	Academic/commercial implementations	Tumor-informed	Usually yes	Ultra-sensitive mutation tracking approach	Very high analytical sensitivity in selected settings	Mainly research/clinical validation use

*Performance characteristics should not be directly compared across platforms because studies differ in population, timing of sampling, assay design, and endpoint definitions.

supplementary specification, additional remarks: Although several ctDNA platforms have demonstrated promising analytical and clinical performance, no universal assay standard has been established. Differences in sequencing technology, bioinformatic pipelines, positivity thresholds, and validation strategies remain important barriers to cross-platform harmonization.

Tumor-informed assays first sequence the primary tumor to identify patient-specific alterations. These alterations are subsequently monitored in plasma using highly sensitive personalized assays (16). These approaches generally provide high analytical specificity and very low detection limits,may be limited by turnaround time and clonal evolution between primary tumors and residual disease (37). Tumor-uninformed (tumor-agnostic) assays analyze plasma directly without requiring prior tumor sequencing. These assays use predefined genomic and/or epigenomic markers to detect ctDNA. These assays are more logistically scalable but may be more susceptible to false-positive results, particularly in the setting of clonal hematopoiesis, and may exhibit lower sensitivity for patient-specific low-frequency variants (38–40). Cross-platform differences in assay design, sensitivity, and bioinformatic filtering remain a major challenge for interpretation and standardization (20, 21).

Clinically, the relevance of ctDNA lies in its potential to identify molecular residual disease earlier and more specifically than conventional tools such as carcinoembryonic antigen (CEA) or radiologic imaging (14). Yet this potential should not be overinterpreted. ctDNA positivity does not necessarily imply inevitable clinical recurrence, and ctDNA negativity does not guarantee cure (11). A biologically and analytically nuanced interpretation is therefore essential when considering ctDNA as a guide for postoperative management (23). The principal characteristics of tumor-informed and tumor-uninformed ctDNA detection methods are compared in **Table 2**.

**Table 2:** Comparison of ctDNA detection methods.

Feature	Tumor-Informed	Tumor-Uninformed
Sensitivity	High	Moderate
Specificity	Very high	Variable
CH interference	Low	High
Turnaround time	Longer	Shorter

## 4 Prognostic significance of postoperative ctDNA in stage II colon cancer

The prognostic value of postoperative ctDNA in resected colon cancer is supported by a growing body of prospective evidence, including studies specifically focused on stage II disease (11). Early work by Tie and colleagues provided proof-of-concept for postoperative ctDNA detection. Their study showed that ctDNA positivity identifies stage II colon cancer patients with markedly increased recurrence risk (15). In that setting, postoperative ctDNA positivity was relatively uncommon but strongly associated with subsequent relapse, establishing ctDNA as a molecular marker of postoperative risk that substantially outperformed conventional clinicopathologic features in effect size (41).

These findings have been reinforced by larger prospective cohorts, including studies embedded within broader colorectal cancer programs (14). In the GALAXY study (CIRCULATE-Japan platform), postoperative ctDNA positivity was closely associated with recurrence risk, with a hazard ratio for disease-free survival of 11.99 (95% CI: 8.83–16.27) in MRD-positive patients (17). Across multiple studies, postoperative ctDNA positivity consistently identifies a high-risk subgroup with worse disease-free outcomes. This association remains significant after adjustment for established clinicopathologic factors (42). Such observations suggest that ctDNA captures a dimension of residual disease biology that is not adequately represented by pathological staging alone (34). At the same time, the relatively low proportion of ctDNA-positive patients in stage II disease highlights an important limitation: recurrence can still occur in patients with initially negative postoperative ctDNA results, reflecting either low tumor shedding, limited assay sensitivity, or biological dormancy of micrometastatic deposits (19).

Serial ctDNA monitoring may further improve postoperative risk stratification. In colorectal cancer cohorts, longitudinal ctDNA detection often precedes radiologic recurrence by several months. This finding supports its potential role in molecular surveillance (36, 43). Sustained ctDNA negativity across serial time points is generally associated with a low risk of recurrence, whereas persistent or newly emergent positivity confers a substantially increased risk (44). However, most of these data derive from mixed-stage populations, and the clinical implications of earlier molecular detection remain uncertain. Earlier detection alone does not necessarily translate into improved survival unless it can be linked to an effective intervention (23, 35).

Although ctDNA demonstrates clear prognostic relevance, its clinical interpretation warrants caution (18). First, ctDNA is not equivalent to viable residual tumor burden and should not be treated as a perfect surrogate for MRD (25). Second, the negative predictive value of postoperative ctDNA is high but not absolute, particularly in low-shedding disease. The biological reasons for this limitation are conceptually summarized in **Figure 2**, which delineates how tumor shedding efficiency, micrometastatic niche characteristics, and assay sensitivity thresholds collectively determine whether residual disease escapes molecular detection. Third, differences in assay design, sampling time, and positivity thresholds limit direct comparison across studies (21). Finally, strong prognostic stratification should not be conflated with predictive utility (20). At present, the most robust conclusion is that postoperative ctDNA constitutes a powerful prognostic biomarker in stage II colon cancer, whereas its ability to identify patients who benefit from a specific adjuvant treatment strategy remains less certain (8).

## 5 ctDNA-guided adjuvant therapy in stage II colon cancer: evidence and unresolved questions

The clinical value of postoperative ctDNA testing lies in its potential to improve adjuvant treatment allocation beyond conventional clinicopathologic criteria (11). In principle, ctDNA-guided management may support two strategies. The first is treatment de-escalation in ctDNA-negative patients to avoid unnecessary chemotherapy. The second is treatment escalation in ctDNA-positive patients to target molecular residual disease before clinical recurrence occurs (36). These two use cases must be clearly distinguished, as the current evidence base is substantially stronger for the former than for the latter (23).

The strongest prospective evidence for ctDNA-guided management in stage II colon cancer comes from the DYNAMIC trial (15). In this randomized study, a ctDNA-guided strategy reduced the proportion of patients receiving adjuvant chemotherapy without compromising recurrence-free survival compared with standard management (41). These results support the feasibility of using postoperative ctDNA to reduce overtreatment in selected patients. However, the interpretation of the DYNAMIC trial warrants appropriate caution. The study was designed as a non-inferiority trial. Therefore, its findings support the safety of a chemotherapy-sparing approach within the predefined trial population and margin. However, the study does not demonstrate the superiority of ctDNA-guided management (45–48). Several limitations should be considered. The number of recurrence events was limited. The trial was conducted within a specific healthcare setting. In addition, the assay used may not be directly interchangeable with other ctDNA platforms.

By contrast, evidence supporting treatment escalation in ctDNA-positive patients remains incomplete and, in part, conflicting (45). Observational studies have suggested that ctDNA-positive patients who receive adjuvant chemotherapy may have better outcomes than those managed without systemic therapy. However, these findings should be interpreted cautiously because of potential confounding by indication, treatment selection bias, and time-related biases such as immortal time bias (48). For example, patients must remain alive and clinically eligible long enough to receive adjuvant therapy, which may introduce bias when comparing treated and untreated groups. Therefore, these observational associations cannot establish that ctDNA positivity predicts benefit from chemotherapy, and prospective randomized biomarker-stratified trials are required to determine whether treatment escalation improves outcomes in ctDNA-positive patients. In an exploratory analysis, the GALAXY study showed that ctDNA-positive patients had a reduced risk of recurrence after adjuvant chemotherapy; however, owing to limitations in study design, a causal relationship could not be established (49). The COBRA trial represents an important example of a neutral interventional study evaluating whether ctDNA-guided escalation could improve outcomes in patients with molecular residual disease. Although the trial did not demonstrate a clear benefit of chemotherapy guided by ctDNA status and was terminated early for futility, these findings should be interpreted cautiously. The relatively small number of ctDNA-positive patients, low event rates, and limited statistical power may have restricted the ability to detect a clinically meaningful treatment effect. Furthermore, ctDNA-positive disease likely represents a biologically heterogeneous population, including patients with different tumor growth kinetics, metastatic potential, and treatment sensitivity.

Therefore, the negative results of COBRA should not be interpreted as evidence that ctDNA lacks clinical relevance, but rather as highlighting that ctDNA positivity alone may be insufficient to define a population that benefits from conventional chemotherapy escalation. Future trials with adequate statistical power, refined patient selection, and biologically informed treatment strategies are required before ctDNA-guided escalation can be incorporated into routine clinical practice (50). One possible explanation is that ctDNA-positive residual disease may be biologically heterogeneous, encompassing lesions with variable growth kinetics, immune control, or chemotherapy sensitivity (19, 34).

Another proposed application of ctDNA is treatment response monitoring during or after adjuvant therapy (43). Clearance of ctDNA has been associated with favorable outcomes in observational analyses, whereas persistent positivity predicts recurrence (17, 41, 51). Although biologically plausible, these associations should not yet be interpreted as evidence that modifying treatment on the basis of ctDNA kinetics improves patient outcomes (14). Analytical variability at very low variant allele fractions, lack of harmonized definitions of ctDNA clearance, and the absence of prospective intervention data limit the current actionability of serial changes.

A brief statement on current guideline positioning is warranted. Although ctDNA is increasingly recognized as a promising biomarker for postoperative risk assessment, most contemporary guidelines do not yet endorse routine ctDNA-guided adjuvant decision-making as a universal standard of care outside carefully selected contexts or clinical trials. The NCCN Guidelines indicate that the routine use of ctDNA in stage II colorectal cancer has not yet established a standard (4, 52). The SEOM-GEMCAD-TTD Guidelines recommend caution when using ctDNA to guide decision-making routinely outside of clinical trials (24). This cautious stance reflects the need for further interventional validation, especially for treatment escalation.

Taken together, the available evidence supports postoperative ctDNA primarily as a tool for risk stratification and for reducing unnecessary chemotherapy exposure in selected patients with stage II colon cancer. Its role in treatment escalation remains investigational, and its predictive value for benefit from specific adjuvant regimens has not been definitively established. At present, ctDNA should be interpreted within a broader clinical framework that includes pathological risk features, MMR/MSI status, comorbidities, patient preferences, and the practical limitations of the assay used.

## 6 ctDNA in relation to clinicopathologic and molecular risk factors

As shown in **Figure 1**, this conceptual framework defines three distinct clinical pathways based on ctDNA status and conventional risk features. First, ctDNA-negative patients without multiple high-risk features may be considered for treatment de-escalation or standard surveillance. Second, patients with discordant risk profiles (e.g., ctDNA-negative but high-risk pathology, or ctDNA-positive with otherwise low-risk features) require individualized clinical interpretation rather than algorithmic decision-making. Third, ctDNA-positive patients represent a high-risk subgroup; however, given the lack of definitive evidence for benefit from treatment escalation, management in this group remains investigational. This structure emphasizes that ctDNA should not be interpreted in isolation but rather within an integrated, context-dependent decision framework.

### 6.1 Established postoperative risk factors in stage II colon cancer

Risk stratification in stage II colon cancer has traditionally relied on clinicopathologic features associated with an increased probability of recurrence. Commonly used high-risk factors include T4 stage, lymphovascular invasion, perineural invasion, bowel obstruction or perforation, poor differentiation, and examination of fewer than 12 lymph nodes (4, 53). In addition, molecular characteristics such as mismatch repair deficiency or microsatellite instability status have major implications for prognosis and treatment relevance in this setting (10). These parameters remain central to postoperative decision-making because they are routinely available, clinically interpretable, and embedded in existing guidelines (24).

However, traditional risk factors have important limitations (8). Most were established as population-level prognostic variables rather than patient-specific indicators of residual disease burden (6). Their effect sizes are modest, interobserver variability may affect pathological assessment, and substantial heterogeneity in outcome persists within each risk category (7). As a result, many patients categorized as high risk do not recur, whereas some patients lacking conventional high-risk features still develop metastatic relapse.

### 6.2 Incremental prognostic value of postoperative ctDNA

Postoperative ctDNA differs conceptually from clinicopathologic risk factors in that it aims to detect molecular evidence of residual disease directly, rather than inferring risk indirectly from tumor morphology. Prospective studies consistently show that postoperative ctDNA positivity is more strongly associated with recurrence than most conventional pathological features (25, 54). This suggests that ctDNA adds clinically meaningful prognostic information beyond standard staging alone.

Nevertheless, ctDNA should not be viewed as a simple replacement for conventional risk assessment. The prevalence of postoperative ctDNA positivity in stage II disease is relatively low, false-negative results remain clinically relevant, and assay performance varies across platforms (16). In addition, established pathological and molecular features continue to inform treatment selection even in the era of ctDNA, particularly because current evidence does not support using ctDNA in isolation to direct all adjuvant decisions (45).

### 6.3 Toward integrated postoperative risk models

The most clinically useful approach will likely integrate ctDNA with clinicopathologic and molecular variables. These approaches should complement rather than replace each other (36). Notably, Loeffler et al. (2025) developed a deep learning (DL)-based model that predicts disease-free survival directly from hematoxylin and eosin (H&E)-stained whole-slide images in colorectal cancer (55). In the GALAXY cohort (n = 1,404), the model stratified patients into a DL-defined high-risk group (n = 304) and a low-risk group (n = 1,100), with a hazard ratio for recurrence of 2.31. Combining the DL score with MRD status further improved risk stratification in both MRD-positive and MRD-negative subgroups (HR: 2.10). Notably, among patients classified as DL high-risk but MRD-negative, adjuvant chemotherapy was associated with significant benefit (HR: 0.49), whereas no benefit was observed in DL low-risk patients (HR: 0.92), suggesting that combined modeling may refine treatment selection beyond ctDNA status alone (55).

A tissue-based stratification analysis from the GALAXY study presented at the 2024 ASCO Annual Meeting reported a composite model integrating a DL-derived biomarker (DoMore-v1-CRC), pathologic staging, and ctDNA-based MRD assessment. This model classified patients into low-, intermediate-, and high-risk categories. Even among ctDNA-negative patients, the high- and intermediate-risk groups exhibited significantly inferior 24-month recurrence-free survival compared with the low-risk group (high vs. low: 80.9% vs. 92.9%; HR: 2.96), highlighting the added value of multimodal integration (56). Future models may incorporate ctDNA alongside T stage, nodal yield, MMR/MSI status, CEA, molecular subtype, and possibly radiologic or digital pathology features to provide a more refined estimate of recurrence risk and treatment relevance (23). A comparison of clinicopathologic and molecular tools for postoperative risk assessment in stage II colon cancer is provided in **Table 3**. 

**Table 3:** Comparison of clinicopathologic and molecular tools for postoperative risk assessment in stage II colon cancer.

Factor / tool	Type of information	Main strengths	Main limitations	Static or dynamic	Direct MRD?	Current role
T stage (T4)	Extent of primary tumor invasion	Routinely available; embedded in guidelines	Does not measure residual disease; heterogeneity within T category	Static	No	Core adjuvant decision-making
Lymphovascular invasion	Vascular/lymphatic dissemination	Widely used; biologically plausible	Interobserver variability; modest effect size	Static	No	Defines high-risk stage II
Perineural invasion	Neural invasion	Indicates aggressive tumor behavior	Variable reporting; not a direct MRD marker	Static	No	Often included in risk assessment
MMR/MSI status	Molecular feature	Clinically established; treatment relevance	Does not detect residual disease directly	Static	No	Standard molecular marker
CEA	Serum tumor marker	Inexpensive; widely available	Limited sensitivity and specificity	Dynamic	Indirect	Adjunctive surveillance
Radiologic imaging	Macroscopic disease	Essential; broadly available	Limited sensitivity for micrometastases	Dynamic	No	Standard follow-up
Postoperative ctDNA	Tumor-derived plasma DNA	Strong prognostic discrimination; dynamic	False-negatives; assay variability; uncertain predictive value	Dynamic	Indirect (closest to MRD)	Promising; not yet universal standard

## 7 Challenges and controversies in clinical implementation

### 7.1 False-negative and false-positive results

A fundamental limitation of ctDNA-based MRD assessment is that assay results reflect both biological and technical factors. A negative result does not completely exclude residual disease because tumor shedding varies among patients and may be influenced by tumor burden, anatomical location, vascularization, and disease dormancy. Conversely, positive results may occasionally reflect non-tumor-derived alterations, particularly in assays without adequate correction for clonal hematopoiesis. As illustrated in **Figure 2**, several biological factors can contribute to false-negative results. These factors include low-shedding tumors, small-volume micrometastases, dormant tumor cell populations, and anatomically isolated disease. For example, peritoneal deposits may be difficult to detect because of limited vascular access (57). This limitation is especially relevant in stage II colon cancer, where residual disease burden is often below the detection threshold of current assays. Current evidence suggests that tumor-informed assays have lower false-positive rates. This advantage is mainly related to their ability to exclude variants derived from clonal hematopoiesis (CH). In contrast, tumor-agnostic approaches appear more susceptible to CH-related interference; even after applying filtering strategies, only approximately 37% of patients have detectable variants suitable for longitudinal monitoring (57). While matched leukocyte sequencing represents an effective strategy to mitigate false-positive results—preventing approximately 40% of false-positive calls in patients with metastatic colorectal cancer (58)—its implementation has not yet been standardized across analytical platforms (59).

### 7.2 Timing of postoperative sampling

The optimal timing for postoperative ctDNA testing remains to be established. The RECIST Working Group has recently evaluated emerging evidence supporting ctDNA as a biomarker of treatment response and emphasized the need for more rigorous standardization, particularly regarding the optimal timing of sample collection and the definition of analytical thresholds for ctDNA detection (60). Sampling too early after surgery may be confounded by perioperative release of non-tumor cfDNA and by the transient biological effects of surgical trauma, whereas delayed sampling may postpone adjuvant decision-making. Many studies have adopted testing approximately four weeks after surgery, but this window has not been universally validated. The ideal timing may also depend on assay type, recovery kinetics, and whether ctDNA is used for single-point decision-making or serial monitoring (59).

### 7.3 Assay heterogeneity and lack of standardization

Clinical interpretation of ctDNA results remains challenging because assay platforms differ in multiple aspects, including sequencing strategy, detection threshold, bioinformatic filtering, and reporting criteria. These differences limit direct comparison across studies and complicate the establishment of universal clinical thresholds. Tumor-informed and tumor-uninformed assays differ in analytical sensitivity, specificity, tissue requirements, turnaround time, vulnerability to clonal hematopoiesis, and practical scalability (21). Tumor-informed approaches generally demonstrate superior sensitivity and specificity; however, their implementation is constrained by the requirement for prior tumor tissue sequencing and the associated higher costs (21). A recent systematic review has comprehensively delineated the differences in clinical application between tumor-informed and tumor-agnostic assays for MRD detection across solid tumors (59). In addition, pre-analytical variables—such as sample collection tubes, processing time, plasma isolation procedures, and sequencing depth—can influence detection outcomes (61). Standardized reporting of assay performance, positivity thresholds, and analytical validation is essential if ctDNA is to be compared meaningfully across studies and translated reliably into routine clinical care (62).

### 7.4 Clinical action thresholds and implementation

Even when ctDNA is detected, the appropriate clinical response is not always clear. It remains uncertain whether all ctDNA-positive patients should receive standard adjuvant chemotherapy, intensified regimens, alternative systemic approaches, or closer surveillance alone (16, 42). Similarly, the degree of reassurance conferred by a negative result depends on the baseline clinical context and on assay performance. Despite the favorable prognosis observed in ctDNA-negative patients, recurrence still occurs in a non-negligible proportion, highlighting the risk of false-negative results and the biological heterogeneity of MRD (63). These issues are particularly important in stage II disease, where the absolute benefit of adjuvant chemotherapy is small and the consequences of both overtreatment and undertreatment are substantial (64).

### 7.5 Cost-effectiveness and health-system implications

The economic feasibility of ctDNA-guided management remains context-dependent. A model-based cost-effectiveness analysis evaluating ctDNA-guided de-escalation of adjuvant chemotherapy in stage III colon cancer reported that, compared with administering adjuvant chemotherapy to all patients, a ctDNA-guided approach is not currently cost-effective. Under the de-escalation strategies examined, an estimated 52%, 61%, and 88% of patients were predicted to receive adjuvant chemotherapy, with corresponding losses of 0.322, 0.237, and 0.034 quality-adjusted life years (QALYs) per patient, respectively. Sensitivity analyses indicated that the ctDNA-guided strategy could become cost-effective only if key ctDNA-related parameters were substantially improved (65). Consistent with these findings, a systematic review of health economic evaluations of ctDNA-based cancer screening reported that, in most scenarios, ctDNA testing is not cost-effective compared with conventional approaches unless specific conditions are met, such as higher adherence rates (66). Formal health-economic evaluation in diverse practice settings therefore remains necessary before broad implementation can be recommended.

Importantly, the feasibility and value of ctDNA-guided management are likely to differ substantially across healthcare systems with varying levels of resources. In high-resource settings, where advanced sequencing platforms, molecular pathology infrastructure, and reimbursement mechanisms are more readily available, ctDNA testing may be integrated into existing precision oncology workflows after appropriate validation. However, even in these settings, the additional costs associated with personalized assays, serial blood collection, bioinformatic analysis, and clinical interpretation remain important considerations.

In low- and middle-resource settings, implementation may be further constrained by limited access to next-generation sequencing facilities, specialized laboratory expertise, quality-control infrastructure, and reimbursement support. These disparities raise concerns regarding equitable access to ctDNA-guided care and highlight the need for scalable, standardized, and cost-efficient assay platforms. Future health-economic evaluations should therefore incorporate regional differences in healthcare infrastructure, treatment costs, patient populations, and resource availability before widespread adoption can be recommended. The key analytical and implementation challenges associated with postoperative ctDNA testing are summarized in **Table 4**.

**Table 4:** Key analytical and implementation challenges for postoperative ctDNA testing in stage II colon cancer.

Challenge	Clinical relevance	Potential consequence	Mitigation strategies	Priority level
Low tumor shedding	Residual disease often below detection threshold in stage II CRC	False-negative ctDNA despite meaningful residual disease	Highly sensitive assays; serial sampling; integrate with clinicopathologic risk	High
Postoperative timing	Early sampling affected by perioperative cfDNA; late sampling delays treatment	Reduced accuracy or delayed adjuvant planning	Standardize sampling windows; validate by assay type	High
Assay heterogeneity	Platforms vary in sensitivity, specificity, tissue requirements	Poor cross-study comparability	Cross-platform validation; standardized reporting; EQA programs	High
Clonal hematopoiesis	Age-related somatic variants can mimic tumor-derived mutations	False-positive calls; inappropriate treatment escalation	Matched leukocyte sequencing; bioinformatic CHIP filtering	High
Pre-analytical variability	Tube type, processing time, storage affect assay performance	Inconsistent detection and reduced reproducibility	Harmonized pre-analytical SOPs	Medium
Lack of positivity thresholds	Binary classification oversimplifies continuous biological variation	Inconsistent recommendations across platforms	Consensus definitions for positivity, indeterminate results, clearance	High
Limited predictive evidence	Prognostic biomarker ≠ treatment-predictive biomarker	Overinterpretation as stand-alone treatment decision tool	Adequately powered randomized biomarker-stratified trials	Critical
Cost and infrastructure	Repeated testing and specialized assays limit access	Unequal implementation; uncertain health-economic value	Formal cost-effectiveness studies; improve assay scalability	Medium

## 8 Ongoing and recently reported clinical trials

Current clinical trials address several distinct questions. First, they evaluate whether ctDNA-negative patients can safely omit adjuvant chemotherapy. Second, they investigate whether ctDNA-positive patients benefit from treatment escalation. Third, they assess whether serial ctDNA monitoring can guide treatment adaptation. Finally, they examine whether different assay platforms provide comparable clinical information. Conflating these questions risks overstating the maturity of the field. The CIRCULATE-III trial stratifies patients into ctDNA-negative and ctDNA-positive cohorts, in which de-escalation and escalation strategies are evaluated independently (67). Similarly, the CIRCULATE-US trial employs the Signatera™ assay for postoperative ctDNA-based risk stratification and incorporates a study design featuring both initial randomization and a second randomization based on serial ctDNA monitoring (68, 69).

Among these, de-escalation in ctDNA-negative patients currently has the strongest clinical support, largely driven by the DYNAMIC trial and supported indirectly by additional prospective programs. The study included 968 evaluable patients with stage III colon cancer and demonstrated that ctDNA-negative patients (n = 702, 72.5%) had significantly superior 3-year recurrence-free survival compared with ctDNA-positive patients (87% vs. 49%; P < 0.001) (69). In contrast, escalation strategies in ctDNA-positive patients remain less certain, with mixed results across studies and limited randomized evidence. This asymmetry is important: a biomarker may be sufficiently informative to spare treatment in some patients without necessarily being able to identify an effective escalation strategy in others.

Several ongoing and recently reported studies are expected to refine these questions further, including trials embedded in the CIRCULATE platform and other prospective cohorts evaluating assay performance, molecular surveillance, and treatment adaptation. However, interpretation will require careful attention to trial design, assay methodology, ctDNA positivity prevalence, and endpoint selection (70). In particular, studies in stage II disease face statistical challenges because postoperative ctDNA positivity is relatively uncommon, limiting power for biomarker-defined subgroup analyses (15).

Future trial design should therefore prioritize: adequate enrichment of ctDNA-positive populations for escalation studies, harmonized assay reporting, long-term survival endpoints, prespecified analyses of pathological and molecular subgroups, and incorporation of patient-reported outcomes. The investigators of the DYNAMIC-III trial explicitly concluded that ctDNA-positive disease requires novel therapeutic strategies, as treatment intensification with conventional chemotherapy did not translate into improved recurrence-free survival (69). The CIRCULATE-US trial incorporates a dedicated second randomization design: patients who convert to ctDNA-positive status during serial monitoring (cohort B) are randomized to receive fluoropyrimidine plus oxaliplatin (FP+Ox) or a more intensified regimen (FP+Ox+I) (68, 69). In the CIRCULATE-III trial, the primary endpoints are 3-year disease-free survival (DFS) in the ctDNA-negative cohort and 3-year DFS in the ctDNA-positive cohort, with a planned follow-up duration of up to 60 months (67). Selected prospective studies and clinical trials relevant to postoperative ctDNA-guided management are summarized in **Table 5**. Additional evidence is provided by real-world postoperative monitoring cohorts (73), the CIRCULATE-Japan platform (74), contemporary reviews of ctDNA-guided adjuvant treatment (75), and the PEGASUS trial (76).

**Table 5:** Selected prospective studies and clinical trials relevant to postoperative ctDNA-guided management in resected colon cancer.

Study	Population	Design	Assay	Main question	Key Finding	Status/Note
Tie et al. (stage II) (15)	Stage II CRC after curative resection	Prospective observational	Tumor-informed	Does postoperative ctDNA identify high-risk patients?	ctDNA positivity strongly associated with recurrence; proof-of-concept for MRD detection	Published; foundational stage II study
DYNAMIC (16, 42)	Stage II CRC	RCT	Tumor-informed	Can ctDNA-guided management reduce adjuvant chemo without compromising outcomes?	ctDNA-guided strategy reduced chemo use; no compromise in RFS; supports de-escalation	Published; strongest randomized evidence
GALAXY (17, 71)	Resectable CRC incl. stage II	Prospective observational platform	Tumor-informed	Prognostic value of postoperative ctDNA; treatment outcome differences?	ctDNA positivity strongly associated with recurrence; predictive benefit unproven	Published; observational; no causal inference
COBRA (72)	Low-risk stage II CRC	RCT interventional	Tumor-uninformed	Do ctDNA-positive patients benefit from adjuvant chemo escalation?	No clear benefit established; highlights uncertainty around escalation	Early termination for futility
CIRCULATE-III (67)	Resected CRC	Randomized platform	Tumor-informed	Can de-escalation/escalation be independently assessed by ctDNA cohort?	Primary endpoints: 3-yr DFS in ctDNA-negative and ctDNA-positive cohorts	Ongoing; 60-month follow-up planned
CIRCULATE-US (68)	Resected CRC	Prospective platform/interventional	Signatera™	Can ctDNA support risk stratification and treatment adaptation?	Cohort B: second randomization for ctDNA-positive patients	Ongoing; North American setting
DYNAMIC-III (69)	Stage III CRC	Randomized phase 2/3	Tumor-informed	Does treatment intensification improve outcomes in ctDNA-positive patients?	Intensification did not improve RFS; ctDNA-positive disease needs alternative strategies	Published 2025

## 9 Future directions

Future ctDNA research in stage II colon cancer should move beyond demonstrating biological feasibility. The priority should be to define clinically actionable applications. First, interventional studies are needed to determine whether ctDNA-positive patients benefit from specific treatment strategies beyond standard fluoropyrimidine-based chemotherapy. This may include biologically selected approaches, alternative systemic regimens, or immunologically informed strategies, although such applications remain exploratory at present.

Second, assay development is likely to move beyond mutation tracking alone. Integration of methylation profiling, fragmentomics, and multi-analyte plasma approaches may enhance sensitivity in low-shedding disease and reduce the false-negative rate that currently constrains postoperative decision-making. At the same time, robust correction for clonal hematopoiesis and improved standardization of pre-analytical workflows will be essential.

Third, ctDNA may contribute to a broader postoperative precision framework rather than functioning as a stand-alone biomarker. Integrated models combining ctDNA with clinicopathologic features, MMR/MSI status, imaging, CEA, and digital pathology may provide more clinically relevant risk stratification than any single modality alone. Computational approaches, including machine-learning and deep-learning models, may facilitate the integration of these complex multimodal datasets; however, several important challenges must be addressed before clinical implementation. First, most currently developed algorithms are derived from retrospective single-center datasets and require prospective, multi-center external validation to confirm robustness and generalizability across different populations, institutions, and laboratory workflows. Second, algorithm performance may be influenced by biases within training datasets, including patient selection bias, underrepresentation of specific demographic or molecular subgroups, variations in pathological assessment, and differences in imaging acquisition protocols. Third, transparent reporting of model architecture, performance metrics, interpretability, and clinical impact is essential to ensure that these tools provide reproducible and clinically meaningful information. Therefore, artificial intelligence-based approaches should be considered complementary tools that require rigorous validation before being incorporated into routine postoperative decision-making.

Finally, the value of ctDNA-guided surveillance and molecular relapse intervention remains to be determined. Earlier detection of recurrence is clinically meaningful only insofar as it leads to interventions that improve outcomes that matter to patients. Future trials should therefore move beyond traditional oncological endpoints, such as recurrence-free survival, and incorporate patient-centered outcomes, including quality of life, treatment-related toxicity, patient-reported outcomes, and treatment burden.

This consideration is particularly important in stage II colon cancer, where the absolute benefit of adjuvant chemotherapy is relatively modest and avoiding unnecessary treatment exposure represents an important clinical goal. For ctDNA-negative patients, successful implementation of de-escalation strategies should be evaluated not only by maintaining oncological safety but also by reducing chemotherapy-associated adverse effects and improving patient experience. Conversely, for ctDNA-positive patients, intensified treatment strategies should demonstrate meaningful clinical benefit without unacceptable toxicity before routine adoption. Future prospective trials should therefore integrate patient-reported outcomes and health-related quality-of-life assessments alongside survival endpoints to provide a more comprehensive evaluation of ctDNA-guided management.

## 10 Discussion

Postoperative ctDNA represents one of the most promising biomarkers currently under investigation for stage II colon cancer. Its major strength is the ability to identify patients at markedly increased risk of recurrence. It also provides additional prognostic information beyond conventional clinicopathologic factors. Current evidence supports its potential role in reducing unnecessary adjuvant chemotherapy exposure in selected patients, but its predictive value for chemotherapy benefit and its role in treatment escalation remain uncertain. Biological heterogeneity in ctDNA shedding, assay variability, false-negative results, and unresolved implementation challenges continue to limit routine clinical adoption.

The conceptual framework shown in **Figure 1** highlights an important evidence gap. The de-escalation pathway for ctDNA-negative patients is increasingly supported by clinical evidence. However, the escalation pathway for ctDNA-positive patients remains insufficiently defined. The decision to escalate, de-escalate, or simply monitor cannot yet be algorithmically determined, underscoring that **Figure 1** represents a conceptual decision-support framework rather than a currently actionable guideline.

At present, ctDNA should be regarded as a powerful but evolving biomarker rather than a fully established clinical decision-making tool. Although postoperative ctDNA provides strong prognostic information, its predictive value for treatment escalation remains uncertain. Clinical implementation requires standardized assay methodologies, prospective validation, and randomized evidence demonstrating improved patient outcomes. Routine clinical implementation requires not only validated assay performance but also demonstrated impact on patient-centered outcomes in well-designed prospective trials.
